# THE EFFECTS OF MODERATE-INTENSITY AEROBIC EXERCISE ON COGNITIVE FUNCTION IN INDIVIDUALS WITH STROKE-INDUCED MILD COGNITIVE IMPAIRMENT: A RANDOMIZED CONTROLLED PILOT STUDY

**DOI:** 10.2340/jrm.v56.33001

**Published:** 2024-07-02

**Authors:** Yuanling HUANG, Haining OU, Weijian ZHAO, Qiang LIN, Yajing XUE, Rui XIA, Zhouchun TAN, Xiaofang ZHAO, Lifang XIONG, Zeqin YAN, Zubin ZHENG, Junbin WEN

**Affiliations:** 1 Department of Rehabilitation, Guangzhou Dongsheng Hospital, Guangzhou, Guangdong, China; 2 Guangdong Provincial Hospital of Chinese Medicine, Guangzhou, Guangdong, China; 3 Guangzhou Medical University, Guangzhou, Guangdong, China; 4 Department of Rehabilitation, the Fifth Affiliated Hospital of Guangzhou Medical University, Guangzhou, Guangdong, China

**Keywords:** stroke, aerobic exercise, cognitive function, work-ing memory

## Abstract

**Objective:**

To assess the impact of moderate-intensity aerobic exercise on working memory in stroke-induced mild cognitive impairment (MCI).

**Design:**

Randomized, double-blind controlled study.

**Subjects and methods:**

Twenty MCI patients from the Fifth Affiliated Hospital of Guangzhou Medical University (December 2021 to February 2023), aged 34–79, 2–12 months post-stroke, were divided into an experimental group (EG) and a control group (CG), each with 10 participants. The EG underwent standard rehabilitation plus 40 minutes of aerobic exercise, while the CG received only standard therapy, 5 times weekly for 2 weeks. Working memory was tested using the n-back task, and overall cognitive function was measured with the MOCA and MMSE Scales before and after the intervention.

**Results:**

The EG showed higher 3-back correctness (71.80 ± 14.53 vs 56.50 ± 13.66), MOCA scores (27.30 ± 1.57 vs 24.00 ± 3.13), and improved visuo-spatial/executive (4.60 ± 0.52 vs 3.30 ± 1.06) and delayed recall (4.30 ± 0.82 vs 3.00 ± 1.56) on the MOCA scale compared with the CG.

**Conclusion:**

Moderate-intensity aerobic exercise may enhance working memory, visuospatial/executive, and delayed recall functions in stroke-induced MCI patients.

LAY ABSTRACT

This study assessed the effects of routine rehabilitation therapy versus moderate-intensity aerobic exercise on working memory in individuals with stroke-induced mild cognitive impairment. The results suggest that moderate-intensity aerobic exercise could serve as an effective therapeutic approach to enhance working memory in this population.

Poststroke cognitive impairment (PSCI) arises from ischaemic stroke, intracerebral haemorrhage, or subarachnoid haemorrhage. It is a prevalent complication following stroke and the second leading cause of cognitive dysfunction. PSCI adversely affects both survival time and quality of life, exceeding the impact of physical disabilities caused by the stroke in terms of discomfort and inconvenience. As a result, PSCI has emerged as a significant focus in both stroke research and clinical practice. Various interventions, including cognitive training, hyperbaric oxygen therapy, repetitive transcranial magnetic stimulation, acupuncture, and aerobic exercise, have shown promise in enhancing cognitive function in individuals affected by stroke (1). Of these, aerobic exercise is particularly notable for its cost-effectiveness, ease of implementation, and minimal adverse effects. Aerobic exercise involves rhythmic, sustained, purposeful, and conscious activity under conditions of adequate oxygen supply. Research suggests that it enhances cognitive function via several mechanisms, such as affecting brain structure volumes, improving cerebral blood flow and oxygenation, enhancing hippocampal synaptic plasticity, modulating neurotrophic factor expression, and stimulating neurogenesis, angiogenesis, and anti-inflammatory responses (2–4).

Research has demonstrated that aerobic exercise positively impacts cognitive function in both healthy elderly and young individuals (5–8). However, research on the effects of aerobic exercise on cognitive function in individuals with stroke-induced mild cognitive impairment (MCI) – a condition characterized by cognitive deficits without impairments in daily living activities – is limited. Furthermore, studies have shown that aerobic exercise enhances executive function (5, 6, 9–12). Although a meta-analysis indicated significant improvements in overall cognitive performance through aerobic exercise, it reported no beneficial effects on cognitive flexibility, working memory, selective attention, or problem-solving abilities (13). Despite these findings, the impact of aerobic exercise on memory functions, particularly working memory (WM) – the crucial function enabling the control of thoughts and reduction of automatic responses – remains inconclusive. Kamijo et al. (15) observed that aerobic exercise increased hit rates and reduced reaction times, suggesting a beneficial effect on WM. In contrast, Stern et al. (5) found no significant improvements in memory function following aerobic exercise. These divergent results may be attributable to the varying intensities of the aerobic exercises performed.

Moderate aerobic exercise has been specifically linked to enhanced cognitive control (16). Research indicates that both moderate- and high-intensity aerobic exercises are more effective than low-intensity exercises in improving cognitive function. Amaya argues that enjoyment derived from exercise promotes sustained engagement, noting that moderate-intensity aerobic exercise increases enjoyment, whereas high-intensity aerobic exercise does not. In clinical settings, individuals with stroke-induced MCI often prioritize physical rehabilitation over cognitive training. Thus, identifying a rehabilitation method that simultaneously improves physical and cognitive functions is paramount. Aerobic exercise emerges as a promising approach, warranting further investigation into its effects on WM. This study aims to examine the effects of moderate-intensity aerobic exercise on cognitive function, specifically WM, in individuals with stroke-induced MCI.

## METHODS

Ethical considerations were thoroughly addressed with the obtainment of approval from the Ethics Committee of the Fifth Affiliated Hospital of Guangzhou Medical University, which granted permission for this study under the approval number GYWY-L2021-77. The research design was established as a single-centre, double-blind, prospective, randomized controlled trial and was formally registered with the China Clinical Trial Registry under the number ChiCTR2200062833 to ensure transparency and accountability. The trial’s double-blind nature ensured that neither the participants nor the researchers were privy to the group assignments. Prior to their participation, all individuals were required to provide their written informed consent, affirming their understanding and voluntary involvement in the study.

### Sample size estimation

For this randomized controlled trial, participants were allocated into 2 groups: the experimental group, which engaged in moderate-intensity aerobic exercise in addition to conventional rehabilitation training, and the control group, which received only the conventional rehabilitation training. The main outcome measure for the trial was identified as the change in the correct response rate on the 2-back test after the intervention period. Preliminary data from prior studies suggested that the average difference in correct response rates on the 2-back test was notably higher in the experimental group (23.50 ± 17.94) compared with the control group (1.75 ± 8.06). Utilizing the GPower 3.1 statistical software (https://www.psychologie.hhu.de/arbeitsgruppen/allgemeine-psychologie-und-arbeitspsychologie/gpower), and setting the significance level at 0.05 (α) and the power at 0.95 (1-β), it was determined that a total of 24 participants, divided evenly with 12 in each group, would be necessary to achieve reliable and valid results.

### Subjects

**Fig. 1:**
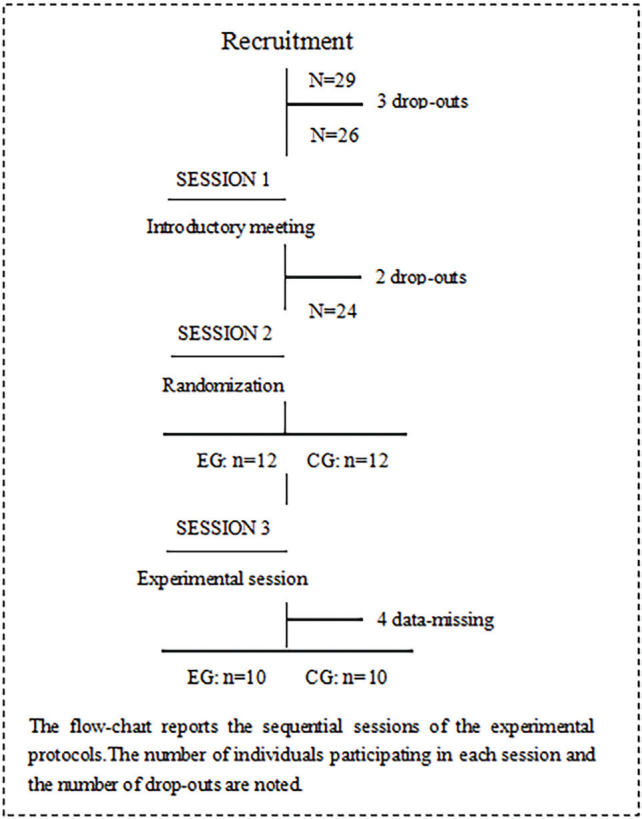
Flowchart of the experimental protocol.

The recruitment phase took place from December 2021 to February 2023 at the Fifth Affiliated Hospital of Guangzhou Medical University. Initially, 29 individuals who had experienced a stroke were considered for inclusion in the trial. However, several candidates were excluded based on specific criteria: 3 for exceeding the age limit of 80 years, 2 for scoring below 18 on the MOCA scale, and 4 due to incomplete data. After these exclusions, the study proceeded with 20 participants (16 males and 4 females), aged between 34 and 79 years, all diagnosed with mild cognitive impairment (MCI). These participants were then randomized in a 1:1 ratio to either the experimental group (EG) or the control group (CG), with each group consisting of 10 participants (8 males and 2 females) (Fig. 1). This study was conducted in strict adherence to the ethical guidelines outlined in the World Medical Association Declaration of Helsinki (2014) (19).

### Enrolment of subjects

*Inclusion criteria*: (*i*) patients diagnosed with stroke according to the 2021 Edition of the Chinese Stroke Prevention and Control Guidelines, confirmed by cranial CT or MRI; (*ii*) patients in a stable condition, aged 34–79 years; (*iii*) patients with no prior cognitive impairment or aphasia, able to participate in all examinations and sign the informed consent form; (*iv*) patients with MoCA scores between 18 and 25 (20), indicative of mild cognitive impairment; (*v*) patients capable of active bicycling and walking with crutches; (*vi*) patients able to complete a cardiopulmonary exercise test.

*Exclusion criteria*: (*i*) patients with severe cardiopulmonary, hepatic, renal, or other significant systemic diseases; (*ii*) patients with severe osteoporosis; (*iii*) patients with a medical history of conditions that could affect cognitive function, such as Parkinson’s disease, traumatic brain injury, or psychiatric disorders; (*iv*) patients currently taking medications known to enhance cognitive function, as specified in the 2021 Expert Consensus on Cognitive Impairment after Stroke (Class I–IIa recommendations, Class A–B evidence), including donepezil and memantine.

### Interventions

**Fig. 2:**
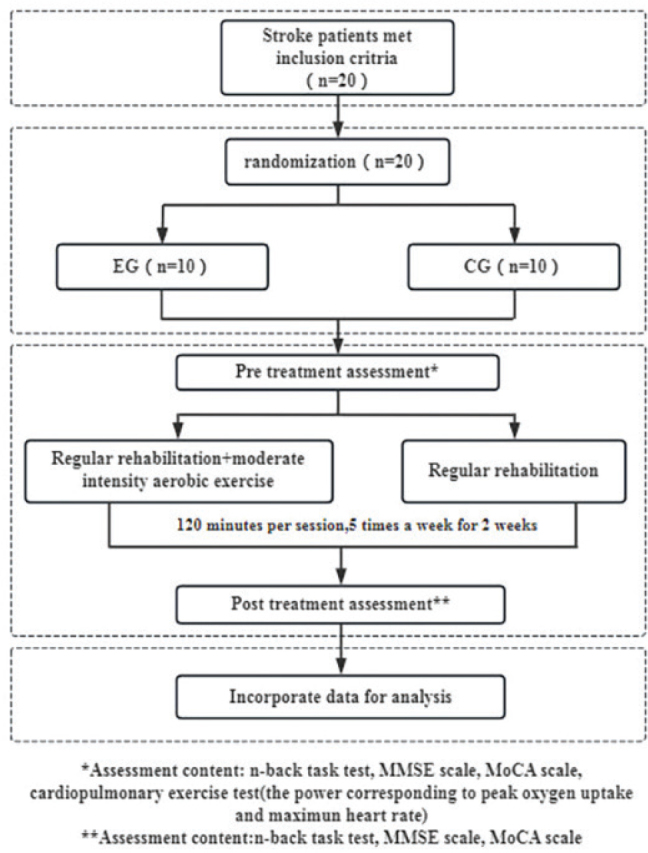
Technical roadmap of the test.

**Fig. 3:**
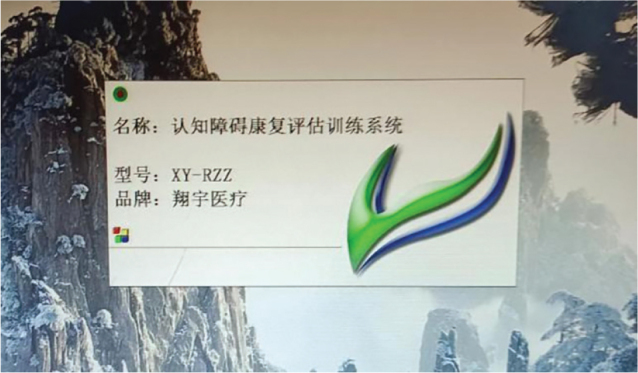
n-back test evaluation system.

**Fig. 4:**
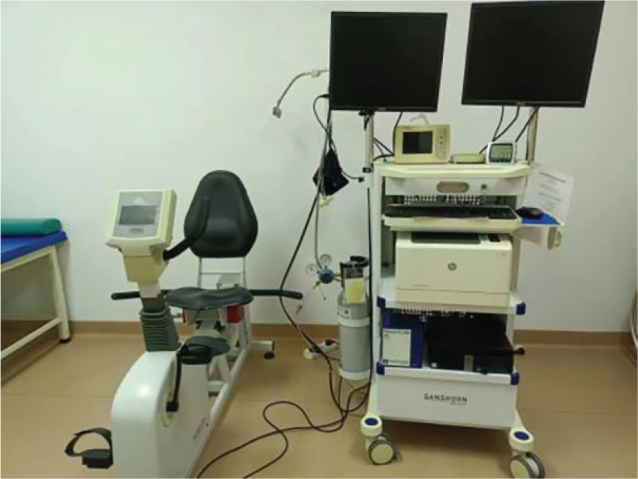
Cardiopulmonary exercise test apparatus.

Before the commencement of the intervention, all participants were thoroughly briefed on the trial process (see Fig. 2), and their informed consent was obtained. Comprehensive assessments were conducted to gather medical history and demographic information, along with measurements of NIHSS scores, height, and weight. Cognitive functions were evaluated using the MMSE scale, MOCA scale, and working memory assessments via the n-back task test, utilizing the Cognitive Impairment Rehabilitation Assessment and Training System (Xiangyu Medical, Anyang, Henan, China; Model: XY-RZZ) (Fig. 3). A cardiopulmonary exercise test was administered using equipment from Kangxun, Germany (model and serial numbers specified) (Fig. 4), to measure each participant’s power load at peak oxygen uptake (PVO_2_) and maximum heart rate (HRmax) on the day they were included in the study. After the 2-week intervention period, all participants were reassessed using the same cognitive scales and task test.

*Control group (CG)*: Participants in the control group underwent routine rehabilitation training, which included physical modality therapy, acupuncture treatment, movement therapy, and occupational therapy. They received 40 minutes of physical modality therapy for limbs and trunk, 30 minutes of acupuncture, 30 minutes of movement therapy, and 20 minutes of occupational therapy, administered 5 times a week for 2 weeks. Movement therapy encompassed turning–transferring training, limb stretching, joint mobilization, balance and coordination training, and walking training. Occupational therapy focused on hand function training and enhancing daily living skills.

*Experimental group (EG)*: Participants in the experimental group participated in moderate-intensity aerobic exercise in addition to their routine rehabilitation training, conducted 5 times a week for 2 weeks.

**Fig. 5:**
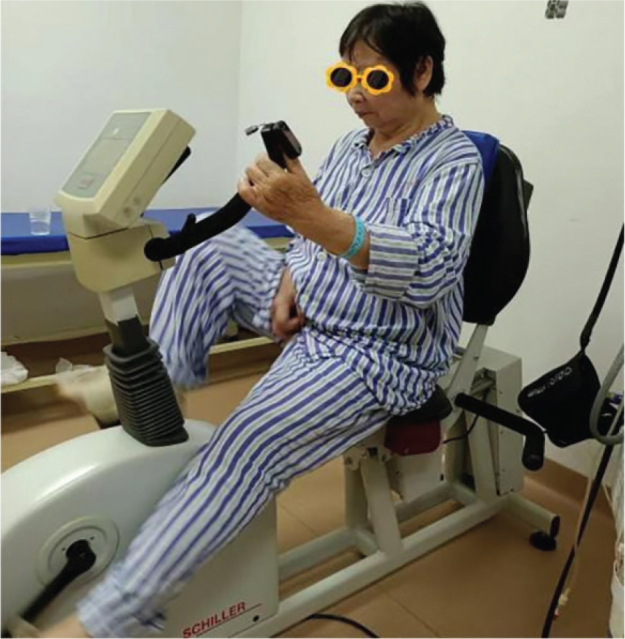
Moderate intensity aerobic exercise.

*Moderate-intensity aerobic exercise*: The aerobic exercise was performed on a rehabilitation treadmill with resistance and power limitations (Fig. 5). Participants had previously undergone cardiopulmonary exercise tests on the day of inclusion to determine the power load at peak oxygen uptake (PVO_2_) and the maximum heart rate (HRmax). The target heart rate for moderate-intensity exercise was set at 65%, the midpoint within the 55–74% range of HRmax, serving as the criterion for exercise intensity. Before starting, participants in the EG familiarized themselves with the treadmill’s use and rules. They then warmed up at 60 RPM for 5 min at 25% of the power load at PVO_2_, followed by 30 min of moderate-intensity exercise at 50% of the power load at PVO_2_. They were instructed to maintain a heart rate at approximately 65% of their HRmax by adjusting the treadmill speed. The session concluded with a 5-min cooldown at 60 RPM at 25% of the power load at PVO_2_. This routine was repeated 5 times weekly for 2 weeks. Throughout the exercise sessions, participants wore oximeters to monitor blood oxygen levels and heart rate.

### Cognitive assessment

*MOCA scale*: The MOCA scale serves as a screening instrument for mild cognitive impairment (MCI). Hong et al. (20) noted that the MoCA total score is influenced by age and education level. For MCI, the use of education-stratified cutoff scores is advised: 18 for those with ≤ 9 years of education, and 22 for those with 9–12 years of education (20). Based on the testing of elderly populations in Guangzhou, China, some researchers have set the cutoff value at 25 points on the MoCA scale. Scores ranging from 14 to 25 are considered indicative of MCI, while scores below 14 suggest dementia (21). The MOCA scale evaluates 8 cognitive domains: visuospatial and executive functions, naming, memory, attention, speech, abstraction, delayed recall, and orientation, with a total possible score of 30. Higher scores denote better cognitive performance. The scale is highly sensitive (80–100%) and moderately specific (50–76%) (22). A total MoCA score below 26 suggests cognitive impairment, while scores between 18 and 25 are indicative of MCI (23). Participants were assessed using the MOCA scale before and after the 2-week intervention period.

*MMSE Scale*: The MMSE scale is a widely utilized tool in clinical settings for screening cognitive impairment. It is moderately sensitive (40–60%) and highly specific (65–90%), known for its ease of use, albeit time-consuming. The scale assesses 7 cognitive domains: time orientation, place orientation, immediate memory, attention and calculation, delayed recall, visuospatial ability, and language, with a total score of 30 (24). Patients with MMSE scores below 27 are considered cognitively impaired, with cutoff points adjusted according to literacy levels. The MMSE scale was administered to all participants before and after the 2-week intervention period.

**Fig. 6:**
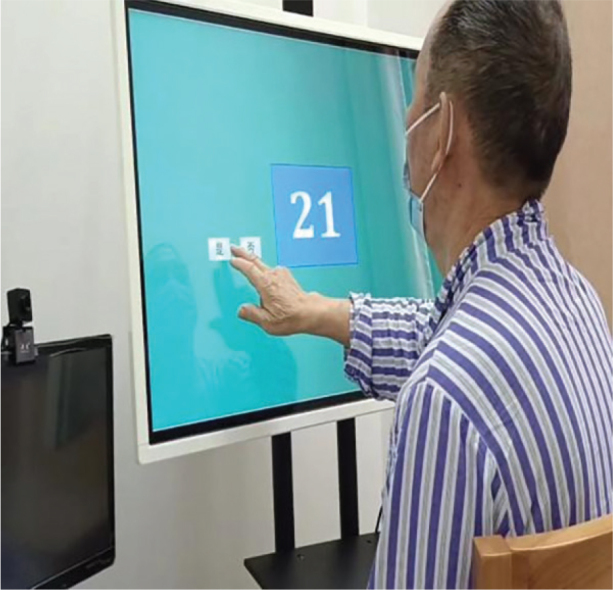
n-back task test.

**Fig. 7:**
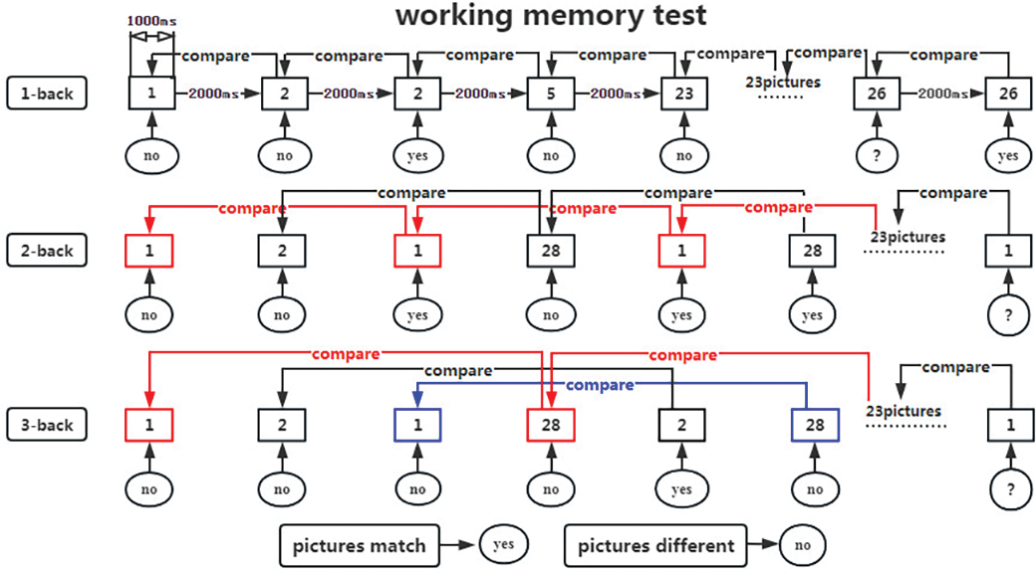
Flowchart of working memory test.

*n-back task test*: Working memory, crucial for processing and storing incoming information, plays a vital role in human learning by interacting with perception, behaviour, and memory. The n-back task test measures working memory by recording correctness and reaction time (RT) (25). The test is conducted using the Cognitive Impairment Rehabilitation Evaluation and Training System (Xiangyu Medical; Model: XY-RZZ). Subjects are presented with a series of numbered pictures (1–30) by the computer and are required to memorize and respond to them by clicking a button as instructed (Fig. 6). Prior to and after 2 weeks of intervention, all participants perform 3 n-back tasks to assess working memory capacity, as shown in the test flowchart (Fig. 7). During the tasks, digital pictures ranging from 1 to 30 are displayed on the computer screen for 1,000 ms each, followed by a reaction time of 2,000 ms. The total time is 3,000 ms a picture for 30 pictures, with a 60% appearance rate of the same digital picture. The computer automatically records the correct rate and reaction time for each group of 30 trials. The 1-back task test involves comparing the current digital picture with the one displayed in the previous step. Participants press the “Yes“ button on the tablet screen when the digital pictures match and the “No“ button when they do not. In the 2-back task test, participants compare the current digital picture with the ones displayed in the first 2 steps of the sequence, pressing the “Yes“ button for a match and the “No“ button for a difference with a 1-picture gap in between. In the 3-back task test, participants compare the current digital picture with those shown in the first 3 steps of the sequence, pressing the “Yes” button for a match and the “No” button for a difference with a 2-picture gap in between. The computer system automatically records the accuracy and average response time upon task completion. To minimize the impact of unfamiliarity with the rules or poor hand–eye coordination, participants are asked to practise 2–3 times before the official test begins.

### Statistical processing

Ultimately, only 20 cases were included for statistical analysis in this study. Data were collected for baseline, demographic, efficacy, and comparative analyses of the experimental and control groups. All statistical analyses were conducted using SPSS 25.0 (IBM Corp, Armonk, NY, USA). Continuous data were described with means and standard deviations, while categorical data were summarized with frequencies and percentages. For normally distributed variables, *t*-tests were applied. For variables not following a normal distribution, non-parametric tests were used. The primary indices for this study were the independent samples *t*-tests comparing clinical data between groups, and paired samples *t*-tests assessing changes in various evaluation indices pre- and post-intervention. Normality tests were performed, revealing that disease duration, post-intervention total MMSE scores, pre- and post-intervention 1-back correctness, post-intervention 1-back RT, post-intervention 2-back correctness, and pre- and post-intervention 3-back RT were not normally distributed; thus, non-parametric tests will be applied. Differences were deemed statistically significant at *p* < 0.05.

## RESULTS

Twenty subjects successfully completed the entire experiment without any side effects or complaints of pain, reflecting the safety and feasibility of the intervention protocols.

### Baseline data

Table I, which presents baseline demographic and clinical data, shows no statistically significant differences between the experimental group (EG) and the control group (CG) in terms of demographic information and various baseline measures such as NIHSS score, MMSE total score, MOCA total score, 1-back correctness, 1-back reaction time (RT), 2-back correctness, 2-back RT, 3-back correctness, 3-back RT, and the cognitive domains assessed by the MOCA scale including visuospatial and executive functions, naming, memory, attention, language, abstraction, delayed recall, and orientation (*p* > 0.05).

**Table I:** Between-group comparison of baseline data

Item	EG (*n* = 10)	CG (*n* = 10)	t/Z	*p*-value
Age	63.80 ± 7.50	58.30 ± 8.10	1.58	0.132
BMI	22.72 ± 2.12	24.61 ± 2.04	–2.03	0.058
MMSE total dcore	28.50 ± 1.58	27.10 ± 2.77	1.39	0.182
MoCA total dcore	22.80 ± 1.69	22.50 ± 3.31	0.26	0.801
1-back RT	1063.90 ± 313.87	1006.10 ± 215.36	9.48	0.637
2-back correctness	59.90 ± 18.27	62.20 ± 14.32	–0.31	0.758
2-back RT	1198.00 ± 312.48	1038.20 ± 476.92	0.89	0.387
3-back correctness	58.90 ± 21.73	55.50 ± 16.47	0.39	0.698
Visuospatial/Executive in MOCA scale	3.90 ± 1.20	3.50 ± 0.85	0.86	0.400
Naming in MOCA scale	3.00 ± 0.00	2.80 ± 0.42	1.50	0.168
Memory in MOCA scale	–	–	–	–
Attention in MOCA scale	5.70 ± 0.48	5.30 ± 1.25	0.94	0.358
Language in MOCA scale	1.30 ± 0.67	1.90 ± 0.74	–1.90	0.074
Abstraction in MOCA scale	1.10 ± 0.57	1.60 ± 0.52	–2.06	0.054
Delayed recall in MOCA scale	2.00 ± 1.63	2.20 ± 1.75	–0.26	0.795
Orientation in MOCA scale	5.80 ± 0.42	5.40 ± 1.35	0.89	0.391
Disease duration	3 (2 ~ 6.5)	2.5 (2 ~ 6.25)	–0.834	0.404
NIHSS score	3.5 (2.75 ~ 4.25)	4 (2.75 ~ 5.25)	–0.048	0.961
1-back correctness	90 (81.5 ~ 94)	88 (79.25 ~ 97)	–1.722	0.085
3-back RT	1092.5 (870.25 ~ 1127)	1178 (1006.75 ~ 1513.5)	–0.851	0.395

EG: Experimental Group, CG: Control Group. *Indicates *p* < 0.05, **indicates *p* < 0.01, where *p* < 0.05 suggests statistical significance.

### Post-intervention outcomes

According to Table II, post-intervention, the EG demonstrated significantly higher scores than the CG in the MOCA total score (*p* < 0.01), 3-back correctness (*p* < 0.05), visuospatial/executive score (*p* < 0.05), and delayed recall score on the MOCA scale (*p* < 0.05).

**Table II:** Between-group comparison of cognitive function after the intervention (consistent with normal distribution)

Item	EG (*n* = 10)	CG (*n* = 10)	t/Z	*p*-value
MoCA total score	27.30 ± 1.57	24.00 ± 3.13	2.98	0.008**
2-back RT	1039.10 ± 146.99	1134.70 ± 281.10	-0.95	0.357
3-back correctness	71.80 ± 14.53	56.50 ± 13.66	2.43	0.026*
Visuospatial/Executive in MOCA scale	4.60 ± 0.52	3.30 ± 1.06	3.49	0.004**
Naming in MOCA scale	2.90 ± 0.32	2.90 ± 0.32	0.00	1.000
Memory in MOCA scale	–	–	–	–
Attention in MOCA scale	5.90 ± 0.32	5.50 ± 1.08	1.12	0.286
Language in MOCA scale	2.10 ± 0.99	2.20 ± 0.42	–0.29	0.773
Abstraction in MOCA scale	1.70 ± 0.67	1.70 ± 0.48	0.00	1.000
Delayed recall in MOCA scale	4.30 ± 0.82	3.00 ± 1.56	2.32	0.032*
Orientation in MOCA scale	5.80 ± 0.63	5.60 ± 0.97	0.55	0.591
MMSE total score	30 (28.75 ~ 30)	28.5 (26 ~ 30)	–0.862	0.389
1-back correctness	93 (90 ~ 97.75)	94.5 (77 ~ 97)	–1.343	0.179
1-back RT	912.5 (803.25 ~ 983)	983 (846.25 ~ 1054)	–1.328	0.184
2-back correctness	83 (72.25 ~ 90.75)	68.5 (42.25 ~ 78.5)	–0.237	0.813
3-back RT	1,107.5 (920 ~ 1,267)	1,170.5 (1,089 ~ 1,423)	–0.378	0.705

EG: Experimental Group, CG: Control Group.

* Indicates *p* < 0.05,

** indicates *p* < 0.01, where *p* < 0.05 suggests statistical significance.

### Changes within the experimental group

Table III reveals that within the EG, there were significant increases from pre-intervention to post-intervention in MMSE total score (*p* < 0.05), MOCA total score (*p* < 0.01), 1-back correctness (*p* < 0.05), 2-back correctness (*p* < 0.05), language score (*p* < 0.05), and delayed recall score (*p* < 0.01) on the MOCA scale. There were no statistical differences in orientation, memory, attention and calculation, recall, naming, retelling, three-step command, reading, writing, and rewriting as assessed by the MMSE scale after the moderate-intensity aerobic exercise (*p* > 0.05).

**Table III:** Changes over time of cognitive function in the experimental group

Item	Pre-intervention	Post-intervention	t/Z	*p*-value
MMSE total score	29 (27.5 ~ 30)	30 (28.5 ~ 30)	–2.271^b^	0.023*
1-back correctness	90 (85 ~ 95)	93 (90 ~ 98.5)	–2.198^b^	0.028*
1-back RT	951 (834.5 ~ 1,115.5)	889 (795.5 ~ 975)	–.981^c^	0.326
2-back correctness	57 (50 ~ 73)	83 (75 ~ 91.5)	–2.194^b^	0.028*
3-back RT	1,077 (850.5 ~ 1,131)	1,060 (889 ~ 1,216.5)	–0.059^b^	0.953
MoCA total score	22.80 ± 1.67	27.30 ± 1.57	–16.75	0.000**
2-back RT	1,198.00 ± 312.48	1,039.10 ± 146.99	1.37	0.204
3-back correctness	58.90 ± 21.73	71.80 ± 14.53	–1.69	0.125
Visuospatial/Executive in MOCA scale	3.90 ± 1.20	4.60 ± 0.52	–1.77	0.111
Naming in MOCA scale	3.00 ± 0.00	2.90 ± 0.32	1.00	0.343
Attention in MOCA scale	5.70 ± 0.48	5.90 ± 0.32	–1.50	0.168
Language in MOCA scale	1.30 ± 0.67	2.10 ± 0.99	–2.75	0.022*
Abstraction in MOCA scale	1.10 ± 0.57	1.70 ± 0.67	–2.25	0.051
Delayed recall in MOCA scale	2.00 ± 1.63	4.20 ± 0.92	–3.97	0.003**
Orientation in MOCA scale	5.80 ± 0.42	5.90 ± 0.32	–1.00	0.343
Orientation in MMSE scale	9.80 ± 0.13	10.00 ± 0.00	–1.50	0.168
Memory in MMSE scale	3.00 ± 0.00	3.00 ± 0.00	–	–
Attention and calculation in MMSE scale	4.60 ± 0.31	4.70 ± 0.30	–1.00	0.343
Recall in MMSE scale	2.50 ± 0.22	2.70 ± 0.21	–1.50	0.168
Naming in MMSE scale	2.00 ± 0.00	2.00 ± 0.00	–	–
Retelling in MMSE scale	0.90 ± 0.10	1.00 ± 0.00	–1.00	0.343
Three-step command in MMSE scale	2.90 ± 0.10	3.00 ± 0.00	–1.00	0.343
Reading in MMSE scale	1.00 ± 0.00	1.00 ± 0.00	–	–
Writing in MMSE scale	1.00 ± 0.00	1.00 ± 0.00	–	–
Rewriting in MMSE scale	0.90 ± 0.10	1.00 ± 0.00	–1.00	0.343

b based on negative rank,

c based on positive rank,

* Indicates *p* < 0.05,

** indicates *p* < 0.01, where *p* < 0.05 suggests statistical significance.

### Changes within the control group

Table IV shows that in the CG, there were no significant changes in MMSE total score, MOCA total score, the correctness and reaction time of 1-back, 2-back, and 3-back tasks, or any cognitive domains of the MOCA and MMSE scales after conventional rehabilitation training (*p* > 0.05).

**Table IV:** Changes over time of cognitive function in the control group

Item	Pre-intervention	Post-intervention	t/Z	*p*-value
MMSE total score	28 (25.5 ~ 29)	28.5 (26 ~ 30)	–1.890^b^	0.059
1-back correctness	88 (79.25 ~ 97)	94.5 (77 ~ 97)	–1.355^b^	0.176
1-back RT	989.5 (834.25 ~ 1,146.75)	983 (846.25 ~ 1,054)	–0.102^c^	0.919
2-back correctness	63 (50.5 ~ 72.5)	68.5 (42.25 ~ 78.5)	–0.059^b^	0.953
3-back RT	1,178 (1,006.75 ~ 1,513.5)	1,170.5 (1,089 ~ 1,423)	–0.561^b^	0.575
MoCA total score	22.50 ± 3.31	24.00 ± 3.13	–2.00	0.076
2-back RT	1,038.20 ± 476.92	1,134.70 ± 281.10	–0.52	0.618
3-back correctness	55.50 ± 16.47	56.50 ± 13.66	–0.24	0.817
Visuospatial/Executive in MOCA scale	3.50 ± 0.85	3.30 ± 1.06	0.43	0.678
Naming in MOCA scale	2.80 ± 0.42	2.90 ± 0.32	–0.56	0.591
Attention in MOCA scale	5.30 ± 1.25	5.50 ± 1.08	–0.69	0.509
Language in MOCA scale	1.90 ± 0.74	2.20 ± 0.42	–1.41	0.193
Abstraction in MOCA scale	1.60 ± 0.52	1.70 ± 0.48	–1.00	0.343
Delayed recall in MOCA scale	2.20 ± 1.75	3.00 ± 1.56	–2.23	0.053
Orientation in MOCA scale	5.40 ± 1.35	5.60 ± 0.97	–0.80	0.443
Orientation in MMSE scale	9.10 ± 1.85	9.40 ± 1.58	–1.96	0.081
Memory in MMSE scale	3.00 ± 0.00	3.00 ± 0.00	–	–
Attention and Calculation in MMSE scale	4.40 ± 1.58	4.40 ± 1.58	0.00	1.000
Recall in MMSE scale	1.80 ± 1.23	2.10 ± 0.99	–1.41	0.193
Naming in MMSE scale	2.00 ± 0.00	2.00 ± 0.00	–	–
Retelling in MMSE scale	1.00 ± 0.00	1.00 ± 0.00	–	–
Three-step command in MMSE scale	3.00 ± 0.00	3.00 ± 0.00	–	–
Reading in MMSE scale	1.00 ± 0.00	1.00 ± 0.00	–	–
Writing in MMSE scale	0.90 ± 0.32	1.00 ± 0.00	–1.00	0.343
Rewriting in MMSE scale	0.90 ± 0.32	0.90 ± 0.32	0.00	1.000

b based on negative rank,

c based on positive rank, *Indicates *p* < 0.05, **indicates *p* < 0.01, where *p* < 0.05 suggests statistical significance.

## DISCUSSION

Many individuals who suffer from stroke experience cognitive impairments linked to their neurological conditions. While some research suggests that aerobic exercise does not enhance memory function in healthy adults (5), studies focusing on individuals with stroke are scarce. Therefore, our study aimed to determine whether moderate-intensity aerobic exercise could more positively affect working memory and other cognitive functions in stroke individuals with mild cognitive impairment (MCI) compared with routine rehabilitation training. Our findings indicate that, relative to the control group, participants in the experimental group exhibited significant improvements in the accuracy of 3-back tasks, MOCA total scores, and visuospatial/executive and delayed recall functions following moderate-intensity aerobic exercise (see Table II). Furthermore, improvements were noted in the accuracy of 1-back and 2-back tasks, MMSE total scores, and MOCA language and delayed recall scores in the experimental group post-intervention (Table III). These results suggest that moderate-intensity aerobic exercise may effectively enhance working memory and other specific cognitive domains in individuals with stroke with MCI.

Our results align with previous findings by Zheng et al. (26), who reported that regular Baduanjin training improved cognitive functions in stroke patients, and Sanchez Bezanilla et al. (27), who found that aerobic exercise could mitigate cognitive impairment in patients with post-stroke cognitive impairment (PSCI). Conversely, our study also observed that routine rehabilitation training did not affect working memory or increase MMSE and MOCA scores in individuals with stroke with MCI, indicating no significant enhancement in cognitive functions. This observation is consistent with the study by Tang et al. (28), which suggested that low-intensity aerobic exercise did not improve cognitive functions in stroke patients with cognitive impairments. During routine rehabilitation, finger pulse oximetry revealed that patients’ heart rates did not reach the levels associated with moderate-intensity aerobic exercise, effectively categorizing it as low-intensity exercise.

In conclusion, our research provides evidence that moderate-intensity aerobic exercise is potentially more effective in improving cognitive function and working memory in individuals with stroke compared with routine rehabilitation training, which is akin to low-intensity aerobic exercise. However, further studies are required to compare the effects of low, medium, and high-intensity aerobic exercises on cognitive function in this population.

### Effect of aerobic exercise on persons with stroke

Research indicates that exercise enhances cognition, as the basal ganglia control fine motor movements during physical activity, forming a feedback loop with the prefrontal cortex that impacts higher brain functions, such as learning or cognition (18). A meta-analysis confirms that aerobic exercise generally improves cognitive function, irrespective of the initial cognitive state (29). Kamijo’s team conducted a study demonstrating that even simple aerobic exercises can benefit working memory in middle-aged males (13). Additionally, systematic reviews have shown that an acute bout of aerobic exercise can boost at least one aspect of cognitive performance in healthy older adults (30). Our study suggests that the cumulative effects of 2 weeks of aerobic exercise could also enhance cognitive functions. This improvement may vary with the degree of cognitive impairment, with earlier interventions likely yielding better outcomes. Lifelong aerobic exercise could lead to sustained enhancements in cognitive functions. However, shorter periods such as once, 2 weeks, 3 months, or 6 months of aerobic exercise may offer only transient cognitive benefits. Aerobic exercise has been recognized as a promising new treatment option for improving cognitive functions and may also serve as an effective non-pharmacological intervention for post-stroke cognitive impairment (31–34). Its affordability, minimal side effects, and simplicity are particularly advantageous, making it popular among patients and their families. Aerobic exercise not only enhances limb function in post-stroke individuals but also improves their cognitive functions. In recent years, as medical rehabilitation garners more attention and demand, many experts and scholars advocate for home-based rehabilitation treatments. Aerobic training, as a straightforward rehabilitation method, is poised to become a principal approach in home rehabilitation for stroke patients.

### Effect of aerobic exercise intensity on persons with stroke

During our experiment, it was observed that stroke patients often could not sustain cycling at an aerobic exercise level with a power load equivalent to 50% of their maximum oxygen uptake, primarily due to limb weakness. This limitation is typically linked to limb dysfunction and poor endurance, rather than to cardiopulmonary issues such as chest tightness or shortness of breath. Consequently, we selected a 50% load of the power corresponding to peak oxygen uptake for aerobic exercise training. Participants were instructed to maintain their heart rate at approximately 65% of their maximum heart rate by adjusting the pedalling speed, ensuring the exercise remained at moderate intensity. The intensity of aerobic exercise is a crucial factor influencing the recovery of cognitive function. A meta-analysis demonstrated that moderate-intensity aerobic exercise favourably impacted cognitive dysfunction in post-stroke patients (13). However, Tang et al. reported that neither high- nor low-intensity aerobic exercise improved cognitive and executive functions in patients with post-stroke cognitive impairment (28). Amaya et al. (18) emphasized the importance of exercise enjoyment for sustained engagement and noted that moderate-intensity aerobic exercise increased enjoyment, whereas high-intensity exercise affected decision-making. Studies have shown that both moderate and high-intensity aerobic exercises are linked to better cognitive improvements compared with low-intensity exercise (17). However, patients with limb weakness and poor endurance often find high-intensity aerobic exercise too challenging to tolerate and maintain. Conversely, low-intensity aerobic exercise may not sufficiently challenge their limb functions. Therefore, for stroke patients who are frail and have limited limb function, moderate-intensity aerobic exercise is deemed more suitable and sustainable. It also presents a higher safety coefficient for these patients. Thus, in this study, moderate-intensity aerobic exercise was the chosen method for training persons with stroke with MCI.

### Relationship between visual space, execution, language, delayed recall, and working memory

Working memory (WM) is critical for transcending reflexive input–output reactions to control our thoughts. It is tasked with the online maintenance and execution control of information, featuring a top-down control mechanism known as “execution” (14). A meta-analysis showed that long-term memory (LTM) for items retained in WM improved significantly, an effect that intensified with longer maintenance durations, reflecting sustained activity in delayed memory maintenance. This effect was particularly strong in visual memory, as indicated by statistical evidence (35). Research has shown that working memory load can influence speech perception, suggesting an asymmetric impact on both low-level auditory encoding and high-level language processing of speech, likely due to attention redistribution under mnemonic load (36). Another study confirmed that attention and working memory exhibit synchronous fluctuations, demonstrated through interconnected continuous attention and working memory tasks (37). Notably, our study found that moderate-intensity aerobic exercise could enhance the working memory of persons with stroke with mild cognitive impairment (MCI). This improvement was reflected in enhanced capabilities in visual space, execution, language, and delayed recall as measured by MOCA scale scores in the experimental group. In summary, improvements in visuospatial or executive functions, language, and delayed recall on the MOCA scale in stroke patients with MCI can be attributed to enhanced working memory capability.

### Prospects and shortcomings of this study

The treatment of post-stroke cognitive dysfunction remains limited, despite an increase in the survival rate of stroke survivors and a corresponding rise in disability rates. Cognitive dysfunction post-stroke adversely impacts the recovery of other functions. Aerobic exercise is favoured due to its low cost, simplicity, minimal side effects, and ease of clinical application. However, the optimal intensity of aerobic exercise is still unclear. It is crucial to assess the effects of different aerobic exercise intensities on the cognitive function of stroke patients. Furthermore, investigating both the immediate effects of acute aerobic exercise and the long-term effects of continuous aerobic exercise could provide significant insights for clinical cognitive rehabilitation.

Our study does present some limitations. First, the sample size was small, and both the duration of the intervention and the follow-up period were brief. There is a need to increase the sample size and extend the duration of interventions to strengthen the validity of our findings. Second, despite monitoring the participants’ heart rates, further clarification of the exercise intensity profile is required, as the recorded heart rates did not reach those typical of moderate-intensity aerobic exercise. Lastly, our inclusion criteria for mild cognitive impairment (MCI) did not consider variations in age and education, which, according to Hong, are factors that influence MoCA total scores (20).
